# Simulating the Fast Prediction Strategy of the Sensorimotor System

**DOI:** 10.3390/biomimetics6010014

**Published:** 2021-02-10

**Authors:** Andrea Biscarini

**Affiliations:** Department of Medicine and Surgery, University of Perugia, 06132 Perugia, Italy; andrea.biscarini@unipg.it

**Keywords:** nervous system, sensory receptor, sensory information, prediction, feedback, nonlinearity

## Abstract

The values of a physiological parameter and its time derivatives, detected at different times by different sensory receptors, are processed by the sensorimotor system to predict the time evolution of the parameter and convey appropriate control commands acting with minimum latency (few milliseconds) from the sensory stimulus. We have derived a power-series expansion (U-expansion) to simulate the fast prediction strategy of the sensorimotor system. Given a time-function f, a time-instant $t_{0}$, and a time-increment τ, the U-expansion enables the calculation of $f\left(t_{0}+\tau \right)$ from $f\left(t_{0}\right)$ and the values $f^{\left(n\right)}\left(t_{n}\right)$ of the derivatives $f^{\left(n\right)}$ of f at arbitrarily different times $t_{n}\;\left(n=1,2,\ldots \right)$, instead of time $t_{0}$ as in the Taylor series. For increments τ significantly greater than the maximum t among the differences $\left|t_{n}-t_{n-1}\right|$, the error associated with truncation of the U-expansion at a given order closely equalizes the error of the corresponding Taylor series (t=0) truncated at the same order. Small values of t and higher values of τ correspond to the high-frequency discharge of sensory neurons and the need for longer-term prediction, respectively. Taking inspiration from the sensorimotor system, the U-expansion can potentially provide an analytical background for the development of algorithms designed for the fast and accurate feedback control of nonlinear systems.

## 1. Introduction

One of the main objectives of basic and applied sciences is to predict the time evolution of a physical system based on its current and previous behavior. This information is often used to regulate the system (i.e., to direct the system towards a specific behavior) through appropriate feedback control commands [1,2]. Notably, the human central nervous system (CNS) integrates and processes a continuous stream of afferent exteroceptive, proprioceptive, and interoceptive information originating from a vast array of sensory receptors (mechanoreceptors, chemoreceptors, photoreceptors, and thermoreceptors) to predict in advance the time evolution of the body’s status and functions (for example, to predict the effects of external perturbations, the effects of the execution of a voluntary task, or the development of internal body conditions that might become harmful) and convey appropriate efferent control commands (for example, to maintain postural equilibrium and orientation, enable coordinated and precise movement patterns, stabilize the visual image on the retinas, and restore and maintain homeostasis) [3,4,5,6]. In many real situations, the constitutive equations that govern the dynamics of a physical system are not exactly known or are too complex to be solved analytically, and approximate numerical methods are needed to arrive at a reliable assessment and effective control of the time evolution of the system [7]. For example, most real-world systems, including the great majority of biological systems, are inherently nonlinear in nature [8,9]. These systems are often governed by nonlinear differential equations and are generally analyzed with the use of numerical methods in the time domain [10,11].

The Taylor series provides a powerful analytical tool for the prediction of the evolution of nonlinear functions. A sufficiently smooth function f of time can usually be expanded into a Taylor series [12] about a time instant $t_{0}$, enabling the calculation of the function at a later time $t_{0}+\tau$ (τ being the time increment from $t_{0}$), given the values of f and its derivatives $f^{\left(n\right)}$ (*n* = 1, 2, …) at time $t_{0}$:

$$f\left(t_{0}+\tau \right)=f\left(t_{0}\right)+\sum_{n=1}^{\infty }f^{\left(n\right)}\left(t_{0}\right)\frac{\tau^{n}}{n!}$$

Truncation of the series at a given order *n* yields an error in the calculation of $f\left(t_{0}+\tau \right)$ (referred to as the remainder), which is an infinitesimal of order higher than $\tau^{n}$ as τ→0 [13]. In fact, the CNS seems to use a similar prediction strategy. Indeed, the sensory receptors of the nervous system can collectively sense the value of a physiological parameter and its time derivatives (up to the third order) and may be directly connected with target motor neurons via large-diameter fast-conducting sensory axons and monosynaptic spinal reflex pathways [14,15,16,17,18,19]. As a result, the efferent response elicited by a sensory stimulus can act with a latency of a few milliseconds [4]. 

In spite of this close analogy, the Taylor series is not directly usable to simulate the sensorimotor control system. Indeed, the afferent sensory information originating from different sensory receptors is not synchronized in time, as it is generally engendered at different instants from different receptors. Each piece of information originating from a sensory receptor travels along a specific sensory neural pathway to reach the processing and integration centers. Thus, the CNS can retrieve a complete map of the receptive fields where the sensory stimuli originated from, together with the corresponding sets of time instants when each sensory stimulus was produced in a specific receptive field. None of the existing models dealing with the control strategy of the sensorimotor system have specifically addressed the problem of the lack of time synchronization within a discrete set of sensory information that conveys to the CNS the value of the successive derivatives of a physiological function [20,21,22,23]. 

The purpose of this study was to develop a computational model that enables a *fast* and *accurate* prediction of the time evolution of a physiological parameter, based on the value of the parameter and the values of its time derivatives detected at different times. To accomplish this goal, we have derived a specific power series expansion, hereafter referred to as the “unsynchronized expansion” (or “U-expansion”, for the sake of brevity). Specifically, given a time function f, a time instant $t_{0}$, and a time increment τ, the U-expansion enables the computation of $f\left(t_{0}+\tau \right)$ from the value $f\left(t_{0}\right)$ and the values $f^{\left(n\right)}\left(t_{n}\right)$ of the derivatives $f^{\left(n\right)}$ of f at arbitrarily different times $t_{n}$ (*n* = 1, 2, …), instead of time $t_{0}$ as in the Taylor series. This new series expansion might constitute an analytical background for the study and simulation of the *fast* prediction strategies of the sensorimotor system, yet the demonstration that the sensorimotor system actually relies on this specific algorithm is beyond the scope of this study. Nevertheless, the U-expansion unveils for the first time a seminal, plausible sensorimotor prediction strategy, which is based on the afferent sensory information that conveys to the CNS the unsynchronized values of the successive derivatives of a physiological function. Most important, the U-expansion can potentially provide an analytical background for the development of numerical algorithms based on high-order finite difference methods and designed for the fast and accurate feedback control of nonlinear systems.

## 2. Materials and Methods

Let us consider the Taylor series expansion of the time function f about the time instant $t_{0}$:

(1) $$f\left(t_{0}+\tau \right)=f\left(t_{0}\right)+f^{\left(1\right)}\left(t_{0}\right)\tau +f^{\left(2\right)}\left(t_{0}\right)\frac{\tau^{2}}{2!}+f^{\left(3\right)}\left(t_{0}\right)\frac{\tau^{3}}{3!}+f^{\left(4\right)}\left(t_{0}\right)\frac{\tau^{4}}{4!}+f^{\left(5\right)}\left(t_{0}\right)\frac{\tau^{5}}{5!}+\ldots =f\left(t_{0}\right)+\sum_{k=1}^{\infty }f^{\left(k\right)}\left(t_{0}\right)\frac{\tau^{k}}{k!}$$

where τ is the time increment from $t_{0}$. To derive the U-expansion, the derivatives $f^{\left(n\right)}\left(t_{0}\right)\;$ in the right-hand side of Equation (1) will be recursively expanded in the Taylor series about a set of arbitrary instants $t_{n}$ (*n* = 1, 2, …), as detailed in the following subsections.

### 2.1. Expansion of the First-Order Term

In the first order-term $f^{\left(1\right)}\left(t_{0}\right)\tau$ of the Taylor series (1), $f^{\left(1\right)}\left(t_{0}\right)$ is expressed as the Taylor series expansion of $f^{\left(1\right)}$ about $t_{1}$ and for a $t_{0}-t_{1}$ increment:

$$f^{\left(1\right)}\left(t_{0}\right)=f^{\left(1\right)}\left(t_{1}\right)+f^{\left(2\right)}\left(t_{1}\right)(t_{0}-t_{1})+f^{\left(3\right)}\left(t_{1}\right)\frac{{\left(t_{0}-t_{1}\right)}^{2}}{2!}+f^{\left(4\right)}\left(t_{1}\right)\frac{{\left(t_{0}-t_{1}\right)}^{3}}{3!}+f^{\left(5\right)}\left(t_{1}\right)\frac{{\left(t_{0}-t_{1}\right)}^{4}}{4!}+\ldots$$

In this equation, $f^{\left(2\right)}\left(t_{1}\right)$ is expressed as the expansion of $f^{\left(2\right)}$ about $t_{2}$ and for a $t_{1}-t_{2}$ increment:

$$\begin{array}{c} f^{\left(1\right)}\left(t_{0}\right)=f^{\left(1\right)}\left(t_{1}\right)+\left(t_{0}-t_{1}\right)\left[f^{\left(2\right)}\left(t_{2}\right)+f^{\left(3\right)}\left(t_{2}\right)\left(t_{1}-t_{2}\right)+f^{\left(4\right)}\left(t_{2}\right)\frac{{\left(t_{1}-t_{2}\right)}^{2}}{2!}+f^{\left(5\right)}\left(t_{2}\right)\frac{{\left(t_{1}-t_{2}\right)}^{3}}{3!}+\ldots \right]\\ \;+f^{\left(3\right)}\left(t_{1}\right)\frac{{\left(t_{0}-t_{1}\right)}^{2}}{2!}+f^{\left(4\right)}\left(t_{1}\right)\frac{{\left(t_{0}-t_{1}\right)}^{3}}{3!}+f^{\left(5\right)}\left(t_{1}\right)\frac{{\left(t_{0}-t_{1}\right)}^{4}}{4!}+\ldots \end{array}$$

Here, the two 3rd-order derivatives are expanded in the Taylor series: $f^{\left(3\right)}\left(t_{1}\right)\;$is expressed as the expansion of $f^{\left(3\right)}$ about $t_{3}$ and for a $t_{1}-t_{3}$ increment, while $f^{\left(3\right)}\left(t_{2}\right)\;$ is again expressed as the expansion of $f^{\left(3\right)}$ about $t_{3}$, but for a *t*_2_ − *t*_3_ increment:

$$\begin{array}{cc} f^{\left(1\right)}\left(t_{0}\right)=f^{\left(1\right)}\left(t_{1}\right) & +\left(t_{0}-t_{1}\right)[f^{\left(2\right)}\left(t_{2}\right)+f^{\left(3\right)}\left(t_{3}\right)\left(t_{1}-t_{2}\;\right)+f^{\left(4\right)}\left(t_{3}\right)\left(t_{1}-t_{2}\;\right)\left(t_{2}-t_{3}\;\right)+f^{\left(5\right)}\left(t_{3}\right)\left(t_{1}-t_{2}\;\right)\frac{{\left(t_{2}-t_{3}\;\right)}^{2}}{2!}\\ & +f^{\left(4\right)}\left(t_{2}\;\right)\frac{{\left(t_{1}-t_{2}\;\right)}^{2}}{2!}+f^{\left(5\right)}\left(t_{2}\;\right)\frac{{\left(t_{1}-t_{2}\;\right)}^{3}}{3!}+\ldots ]\\ & +\frac{{\left(t_{0}-t_{1}\right)}^{2}}{2!}\left[f^{\left(3\right)}\left(t_{3}\right)+f^{\left(4\right)}\left(t_{3}\right)\left(t_{1}-t_{3}\right)+f^{\left(5\right)}\left(t_{3}\right)\frac{{\left(t_{1}-t_{3}\right)}^{2}}{2!}+\;\cdots \right]+f^{\left(4\right)}\left(t_{1}\right)\frac{{\left(t_{0}-t_{1}\right)}^{3}}{3!}\\ & +f^{\left(5\right)}\left(t_{1}\right)\frac{{\left(t_{0}-t_{1}\right)}^{4}}{4!}+\ldots \end{array}$$

The three 4th-order derivatives $\;f^{\left(4\right)}\left(t_{1}\right)$, $f^{\left(4\right)}\left(t_{2}\right)$, and $f^{\left(4\right)}\left(t_{3}\right)$ are then expressed as the expansion of $f^{\left(4\right)}$ about $t_{4}$, for increments given by $t_{1}-t_{4},\;t_{2}-t_{4}$, and $t_{3}-t_{4}$, respectively:

$$\begin{array}{cc} f^{\left(1\right)}\left(t_{0}\right)=f^{\left(1\right)}\left(t_{1}\right) & +\left(t_{0}-t_{1}\right)[f^{\left(2\right)}\left(t_{2}\right)+f^{\left(3\right)}\left(t_{3}\right)\left(t_{1}-t_{2}\right)+f^{\left(4\right)}\left(t_{4}\right)\left(t_{1}-t_{2}\right)\left(t_{2}-t_{3}\right)\\ & +f^{\left(5\right)}\left(t_{4}\right)\left(t_{1}-t_{2}\right)\left(t_{2}-t_{3}\right)\left(t_{3}-t_{4}\right)+f^{\left(5\right)}\left(t_{3}\right)\left(t_{1}-t_{2}\right)\frac{{\left(t_{2}-t_{3}\right)}^{2}}{2!}+f^{\left(4\right)}\left(t_{4}\right)\frac{{\left(t_{1}-t_{2}\right)}^{2}}{2!}\\ & +f^{\left(5\right)}\left(t_{4}\right)\frac{{\left(t_{1}-t_{2}\right)}^{2}}{2!}\left(t_{2}-t_{4}\right)+f^{\left(5\right)}\left(t_{2}\right)\frac{{\left(t_{1}-t_{2}\right)}^{3}}{3!}+\ldots ]\\ & +\frac{{\left(t_{0}-t_{1}\right)}^{2}}{2!}\left[f^{\left(3\right)}\left(t_{3}\right)+f^{\left(4\right)}\left(t_{4}\right)\left(t_{1}-t_{3}\right)+f^{\left(5\right)}\left(t_{4}\right)\left(t_{1}-t_{3}\right)\left(t_{3}-t_{4}\right)+f^{\left(5\right)}\left(t_{3}\right)\frac{{\left(t_{1}-t_{3}\right)}^{2}}{2!}+\ldots \right]\\ & +\frac{{\left(t_{0}-t_{1}\right)}^{3}}{3!}\left[f^{\left(4\right)}\left(t_{4}\right)+f^{\left(5\right)}\left(t_{4}\right)\left(t_{1}-t_{4}\right)\right]+f^{\left(5\right)}\left(t_{1}\right)\frac{{\left(t_{0}-t_{1}\right)}^{4}}{4!}+\ldots \end{array}$$

The process continues by expressing the four 5th-order derivatives $f^{\left(5\right)}\left(t_{1}\right)$, $f^{\left(5\right)}\left(t_{2}\right)$, $f^{\left(5\right)}\left(t_{3}\right)$, and $f^{\left(5\right)}\left(t_{4}\right)$ as the expansion of $f^{\left(5\right)}$ about $t_{5}$, for increments given by $t_{1}-t_{5}$, $t_{2}-t_{5}$, $t_{3}-t_{5}$, and $t_{4}-t_{5}$, respectively. With the progressive expansion of higher-order derivatives and grouping together of derivatives of the same order, one gets the following final equation for $f^{\left(1\right)}\left(t_{0}\right)$ (where terms up to the 6th order have been included):

(2) $$\begin{array}{cc} f^{\left(1\right)}\left(t_{0}\right)=f^{\left(1\right)}\left(t_{1}\right) & +f^{\left(2\right)}\left(t_{2}\right)\left(t_{0}-t_{1}\right)+f^{\left(3\right)}\left(t_{3}\right)\left[\frac{{\left(t_{0}-t_{1}\right)}^{2}}{2!}+\left(t_{0}-t_{1}\right)\left(t_{1}-t_{2}\right)\right]\\ & +f^{\left(4\right)}\left(t_{4}\right)\left[\frac{{\left(t_{0}-t_{1}\right)}^{3}}{3!}+\left(t_{0}-t_{1}\right)\frac{{\left(t_{1}-t_{2}\right)}^{2}}{2!}+\frac{{\left(t_{0}-t_{1}\right)}^{2}}{2!}\left(t_{1}-t_{3}\right)+\left(t_{0}-t_{1}\right)\left(t_{1}-t_{2}\right)\left(t_{2}-t_{3}\right)\right]\\ & +f^{\left(5\right)}\left(t_{5}\right)[\frac{{\left(t_{0}-t_{1}\right)}^{4}}{4!}+\left(t_{0}-t_{1}\right)\frac{{\left(t_{1}-t_{2}\right)}^{3}}{3!}+\frac{{\left(t_{0}-t_{1}\right)}^{2}}{2!}\frac{{\left(t_{1}-t_{3}\right)}^{2}}{2!}+\frac{{\left(t_{0}-t_{1}\right)}^{3}}{3!}\left(t_{1}-t_{4}\right)\\ & +\left(t_{0}-t_{1}\right)\left(t_{1}-t_{2}\right)\frac{{\left(t_{2}-t_{3}\right)}^{2}}{2!}+\left(t_{0}-t_{1}\right)\frac{{\left(t_{1}-t_{2}\right)}^{2}}{2!}\left(t_{2}-t_{4}\right)+\frac{{\left(t_{0}-t_{1}\right)}^{2}}{2!}\left(t_{1}-t_{3}\right)\left(t_{3}-t_{4}\right)\\ & +\left(t_{0}-t_{1}\right)\left(t_{1}-t_{2}\right)\left(t_{2}-t_{3}\right)\left(t_{3}-t_{4}\right)]\\ & +f^{\left(6\right)}\left(t_{6}\right)[\frac{{\left(t_{0}-t_{1}\right)}^{5}}{5!}+\left(t_{0}-t_{1}\right)\frac{{\left(t_{1}-t_{2}\right)}^{4}}{4!}+\frac{{\left(t_{0}-t_{1}\right)}^{2}}{2!}\frac{{\left(t_{1}-t_{3}\right)}^{3}}{3!}+\frac{{\left(t_{0}-t_{1}\right)}^{3}}{3!}\frac{{\left(t_{1}-t_{4}\right)}^{2}}{2!}\\ & +\frac{{\left(t_{0}-t_{1}\right)}^{4}}{4!}\left(t_{1}-t_{5}\right)+\left(t_{0}-t_{1}\right)\left(t_{1}-t_{2}\right)\frac{{\left(t_{2}-t_{3}\right)}^{3}}{3!}+\left(t_{0}-t_{1}\right)\frac{{\left(t_{1}-t_{2}\right)}^{2}}{2!}\frac{{\left(t_{2}-t_{4}\right)}^{2}}{2!}\\ & +\left(t_{0}-t_{1}\right)\frac{{\left(t_{1}-t_{2}\right)}^{3}}{3!}\left(t_{2}-t_{5}\right)+\frac{{\left(t_{0}-t_{1}\right)}^{2}}{2!}\left(t_{1}-t_{3}\right)\frac{{\left(t_{3}-t_{4}\right)}^{2}}{2!}+\frac{{\left(t_{0}-t_{1}\right)}^{2}}{2!}\frac{{\left(t_{1}-t_{3}\right)}^{2}}{2!}\left(t_{3}-t_{5}\right)\\ & +\frac{{\left(t_{0}-t_{1}\right)}^{3}}{3!}\left(t_{1}-t_{4}\right)\left(t_{4}-t_{5}\right)+\left(t_{0}-t_{1}\right)\left(t_{1}-t_{2}\right)\left(t_{2}-t_{3}\right)\frac{{\left(t_{3}-t_{4}\right)}^{2}}{2!}\\ & +\left(t_{0}-t_{1}\right)\left(t_{1}-t_{2}\right)\frac{{\left(t_{2}-t_{3}\right)}^{2}}{2!}\left(t_{3}-t_{5}\right)+\left(t_{0}-t_{1}\right)\frac{{\left(t_{1}-t_{2}\right)}^{2}}{2!}\left(t_{2}-t_{4}\right)\left(t_{4}-t_{5}\right)\\ & +\frac{{\left(t_{0}-t_{1}\right)}^{2}}{2!}\left(t_{1}-t_{3}\right)\left(t_{3}-t_{4}\right)\left(t_{4}-t_{5}\right)+\left(t_{0}-t_{1}\right)\left(t_{1}-t_{2}\right)\left(t_{2}-t_{3}\right)\left(t_{3}-t_{4}\right)\left(t_{4}-t_{5}\right)]+\cdots \end{array}$$

Each term in this equation is the product of a number of factors equal to the number of expansions carried out.

### 2.2. Expansion of the Second-Order Term

The step procedure is repeated for the 2nd-order derivative $f^{\left(2\right)}\left(t_{0}\right)$ in the Taylor series (1). Specifically, $f^{\left(2\right)}\left(t_{0}\right)$ is expressed as the Taylor series expansion of $f^{\left(2\right)}$ about $t_{2}$ and for a $t_{0}-t_{2}$ increment:

$$f^{\left(2\right)}\left(t_{0}\right)=f^{\left(2\right)}\left(t_{2}\right)+f^{\left(3\right)}\left(t_{2}\right)\left(t_{0}-t_{2}\right)+f^{\left(4\right)}\left(t_{2}\right)\frac{{\left(t_{0}-t_{2}\right)}^{2}}{2!}+f^{\left(5\right)}\left(t_{2}\right)\frac{{\left(t_{0}-t_{2}\right)}^{3}}{3!}+f^{\left(6\right)}\left(t_{2}\right)\frac{{\left(t_{0}-t_{2}\right)}^{4}}{4!}+\ldots$$

In this equation, $f^{\left(3\right)}\left(t_{2}\right)$ is expressed as the Taylor series expansion of $f^{\left(3\right)}$ about $t_{3}$ and for a $t_{2}-t_{3}$ increment:

$$\begin{array}{cc} f^{\left(2\right)}\left(t_{0}\right)=f^{\left(2\right)}\left(t_{2}\right) & +\left(t_{0}-t_{2}\right)\left[f^{\left(3\right)}\left(t_{3}\right)+f^{\left(4\right)}\left(t_{3}\right)\left(t_{2}-t_{3}\right)+f^{\left(5\right)}\left(t_{3}\right)\frac{{\left(t_{2}-t_{3}\right)}^{2}}{2!}+f^{\left(6\right)}\left(t_{3}\right)\frac{{\left(t_{2}-t_{3}\right)}^{3}}{3!}\cdots \right]\\ & +f^{\left(4\right)}\left(t_{2}\right)\frac{{\left(t_{0}-t_{2}\right)}^{2}}{2!}+f^{\left(5\right)}\left(t_{2}\right)\frac{{\left(t_{0}-t_{2}\right)}^{3}}{3!}+f^{\left(6\right)}\left(t_{2}\right)\frac{{\left(t_{0}-t_{2}\right)}^{4}}{4!}+\cdots \end{array}$$

The two 4th-order derivatives $f^{\left(4\right)}\left(t_{2}\right)$ and $f^{\left(4\right)}\left(t_{3}\right)$ are then expressed as the expansion of $f^{\left(4\right)}$ about $t_{4}$, for increments given by $t_{2}-t_{4}$ and $t_{3}-t_{4}$, respectively:

$$\begin{array}{cc} f^{\left(2\right)}\left(t_{0}\right)=f^{\left(2\right)}\left(t_{2}\right) & +\left(t_{0}-t_{2}\right)[f^{\left(3\right)}\left(t_{3}\right)+f^{\left(4\right)}\left(t_{4}\right)\left(t_{2}-t_{3}\right)+f^{\left(5\right)}\left(t_{4}\right)\left(t_{2}-t_{3}\right)\left(t_{3}-t_{4}\right)+f^{\left(6\right)}\left(t_{4}\right)\left(t_{2}-t_{3}\right)\frac{{\left(t_{3}-t_{4}\right)}^{2}}{2!}\\ & +f^{\left(5\right)}\left(t_{3}\right)\frac{{\left(t_{2}-t_{3}\right)}^{2}}{2!}+f^{\left(6\right)}\left(t_{3}\right)\frac{{\left(t_{2}-t_{3}\right)}^{3}}{3!}\cdots ]\\ & +\frac{{\left(t_{0}-t_{2}\right)}^{2}}{2!}\left[f^{\left(4\right)}\left(t_{4}\right)+f^{\left(5\right)}\left(t_{4}\right)\left(t_{2}-t_{4}\right)+f^{\left(6\right)}\left(t_{4}\right)\frac{{\left(t_{2}-t_{4}\right)}^{2}}{2}\cdots \right]+f^{\left(5\right)}\left(t_{2}\right)\frac{{\left(t_{0}-t_{2}\right)}^{3}}{3!}\\ & +f^{\left(6\right)}\left(t_{2}\right)\frac{{\left(t_{0}-t_{2}\right)}^{4}}{4!}+\cdots \end{array}$$

In the next step, the three 5th-order derivatives $f^{\left(5\right)}\left(t_{2}\right)$, $f^{\left(5\right)}\left(t_{3}\right)$, and $f^{\left(5\right)}\left(t_{4}\right)$ are expressed as the expansion of $f^{\left(5\right)}$ about $t_{5}$, for increments given by $t_{2}-t_{5}$, $t_{3}-t_{5}$, and $t_{4}-t_{5}$, respectively:

$$\begin{array}{cc} f^{\left(2\right)}\left(t_{0}\right)=f^{\left(2\right)}\left(t_{2}\right) & +\left(t_{0}-t_{2}\right)[f^{\left(3\right)}\left(t_{3}\right)+f^{\left(4\right)}\left(t_{4}\right)\left(t_{2}-t_{3}\right)+f^{\left(5\right)}\left(t_{5}\right)\left(t_{2}-t_{3}\right)\left(t_{3}-t_{4}\right)\\ & +f^{\left(6\right)}\left(t_{5}\right)\left(t_{2}-t_{3}\right)\left(t_{3}-t_{4}\right)\left(t_{4}-t_{5}\right)+f^{\left(6\right)}\left(t_{4}\right)\left(t_{2}-t_{3}\right)\frac{{\left(t_{3}-t_{4}\right)}^{2}}{2!}+f^{\left(5\right)}\left(t_{5}\right)\frac{{\left(t_{2}-t_{3}\right)}^{2}}{2!}\\ & +f^{\left(6\right)}\left(t_{3}\right)\frac{{\left(t_{2}-t_{3}\right)}^{2}}{2!}\left(t_{3}-t_{5}\right)+f^{\left(6\right)}\left(t_{3}\right)\frac{{\left(t_{2}-t_{3}\right)}^{3}}{3!}\cdots ]\\ & +\frac{{\left(t_{0}-t_{2}\right)}^{2}}{2!}\left[f^{\left(4\right)}\left(t_{4}\right)+f^{\left(5\right)}\left(t_{5}\right)\left(t_{2}-t_{4}\right)+f^{\left(6\right)}\left(t_{5}\right)\left(t_{2}-t_{4}\right)\left(t_{4}-t_{5}\right)+f^{\left(6\right)}\left(t_{4}\right)\frac{{\left(t_{2}-t_{4}\right)}^{2}}{2}\cdots \right]\\ & +\frac{{\left(t_{0}-t_{2}\right)}^{3}}{3!}\left[f^{\left(5\right)}\left(t_{5}\right)+f^{\left(6\right)}\left(t_{5}\right)\left(t_{2}-t_{5}\right)\right]+f^{\left(6\right)}\left(t_{2}\right)\frac{{\left(t_{0}-t_{2}\right)}^{4}}{4!}+\cdots \end{array}$$

With the progressive expansion of the higher-order derivatives and grouping together of derivatives of the same order, one arrives at the following final equation for $f^{\left(2\right)}\left(t_{0}\right)$ (where terms up to the 6th order have been included):

(3) $$\begin{array}{cc} f^{\left(2\right)}\left(t_{0}\right)=f^{\left(2\right)}\left(t_{2}\right) & +f^{\left(3\right)}\left(t_{3}\right)\left(t_{0}-t_{2}\right)+f^{\left(4\right)}\left(t_{4}\right)\left[\frac{{\left(t_{0}-t_{2}\right)}^{2}}{2!}+\left(t_{0}-t_{2}\right)\left(t_{2}-t_{3}\right)\right]\\ & +f^{\left(5\right)}\left(t_{5}\right)\left[\frac{{\left(t_{0}-t_{2}\right)}^{3}}{3!}+\left(t_{0}-t_{2}\right)\frac{{\left(t_{2}-t_{3}\right)}^{2}}{2!}+\frac{{\left(t_{0}-t_{2}\right)}^{2}}{2!}\left(t_{2}-t_{4}\right)+\left(t_{0}-t_{2}\right)\left(t_{2}-t_{3}\right)\left(t_{3}-t_{4}\right)\right]\\ & +f^{\left(6\right)}\left(t_{6}\right)[\frac{{\left(t_{0}-t_{2}\right)}^{4}}{4!}+\left(t_{0}-t_{2}\right)\frac{{\left(t_{2}-t_{3}\right)}^{3}}{3!}+\frac{{\left(t_{0}-t_{2}\right)}^{2}}{2!}\frac{{\left(t_{2}-t_{4}\right)}^{2}}{2!}+\frac{{\left(t_{0}-t_{2}\right)}^{3}}{3!}\left(t_{2}-t_{5}\right)\\ & +\left(t_{0}-t_{2}\right)\left(t_{2}-t_{3}\right)\frac{{\left(t_{3}-t_{4}\right)}^{2}}{2!}+\left(t_{0}-t_{2}\right)\frac{{\left(t_{2}-t_{3}\right)}^{2}}{2!}\left(t_{3}-t_{5}\right)+\frac{{\left(t_{0}-t_{2}\right)}^{2}}{2!}\left(t_{2}-t_{4}\right)\left(t_{4}-t_{5}\right)\\ & +\left(t_{0}-t_{2}\right)\left(t_{2}-t_{3}\right)\left(t_{3}-t_{4}\right)\left(t_{4}-t_{5}\right)]+\cdots \end{array}$$

### 2.3. Expansion of the Third-Order Term

The procedure progresses with the higher-order derivatives. For example, $f^{\left(3\right)}\left(t_{0}\right)$ is expressed as the Taylor series expansion of $f^{\left(3\right)}$ about $t_{3}$ and for a $t_{0}-t_{3}$ increment:

$$f^{\left(3\right)}\left(t_{0}\right)=f^{\left(3\right)}\left(t_{3}\right)+f^{\left(4\right)}\left(t_{3}\right)\left(t_{0}-t_{3}\right)+f^{\left(5\right)}\left(t_{3}\right)\frac{{\left(t_{0}-t_{3}\right)}^{2}}{2!}+f^{\left(6\right)}\left(t_{3}\right)\frac{{\left(t_{0}-t_{3}\right)}^{3}}{3!}+\cdots$$

$f^{\left(4\right)}\left(t_{3}\right)$ as the Taylor series expansion of $f^{\left(4\right)}$ about $t_{4}$ and for a $t_{3}-t_{4}$ increment:

$$\begin{array}{cc} f^{\left(3\right)}\left(t_{0}\right)=f^{\left(3\right)}\left(t_{3}\right) & +\left(t_{0}-t_{3}\right)\left[f^{\left(4\right)}\left(t_{4}\right)+f^{\left(5\right)}\left(t_{4}\right)\left(t_{3}-t_{4}\right)+f^{\left(6\right)}\left(t_{4}\right)\frac{{\left(t_{3}-t_{4}\right)}^{2}}{2}+\ldots \right]+f^{\left(5\right)}\left(t_{3}\right)\frac{{\left(t_{0}-t_{3}\right)}^{2}}{2!}\\ & +f^{\left(6\right)}\left(t_{3}\right)\frac{{\left(t_{0}-t_{3}\right)}^{3}}{3!}+\cdots \end{array}$$

$f^{\left(5\right)}\left(t_{3}\right)$ and $f^{\left(5\right)}\left(t_{4}\right)$ as the expansion of $f^{\left(5\right)}$ about $t_{5}$, for increments given by $t_{3}-t_{5}$ and $t_{4}-t_{5}$, respectively, and so on, to finally get

(4) $$\begin{array}{cc} f^{\left(3\right)}\left(t_{0}\right)=f^{\left(3\right)}\left(t_{3}\right) & +f^{\left(4\right)}\left(t_{4}\right)\left(t_{0}-t_{3}\right)+f^{\left(5\right)}\left(t_{5}\right)\left[\frac{{\left(t_{0}-t_{3}\right)}^{2}}{2!}+\left(t_{0}-t_{3}\right)\left(t_{3}-t_{4}\right)\right]\\ & +f^{\left(6\right)}\left(t_{6}\right)\left[\frac{{\left(t_{0}-t_{3}\right)}^{3}}{3!}+\left(t_{0}-t_{3}\right)\frac{{\left(t_{3}-t_{4}\right)}^{2}}{2}+\frac{{\left(t_{0}-t_{3}\right)}^{2}}{2!}\left(t_{3}-t_{5}\right)+\left(t_{0}-t_{3}\right)\left(t_{3}-t_{4}\right)\left(t_{4}-t_{5}\right)\right]+\cdots \end{array}$$

### 2.4. The U-Expansion

The procedure outlined in the previous sections highlights that each of the derivatives $f^{\left(k\right)}\left(t_{0}\right)$ in the Taylor series (1) can be expressed in terms of the derivatives $f^{\left(n\right)}\left(t_{n}\right)\;\left(n=k,\;k+1,\;k+2,\;\ldots \right)$ according to the following equation:

(5) $$f^{\left(k\right)}\left(t_{0}\right)=\sum_{n=k}^{\infty }f^{\left(n\right)}\left(t_{n}\right)p_{nk}\;\;\;\;\;\;\;\;\;\;\;\;\;\;\;\;\;\;\;\;\;\;\;\;\;\;\left(k=1,\;2,\;3,\ldots \right)$$

where the $p_{nk}$ coefficients will be readily deduced, in the next section, from Equations (2)–(4). Replacing the first and the higher-order time derivatives $f^{\left(k\right)}\left(t_{0}\right)$ in the right-hand side of the Taylor series (1) with the corresponding expressions given by Equation (5), one gets

$$f\left(t_{0}+\tau \right)=f\left(t_{0}\right)+\sum_{k=1}^{\infty }\left(\frac{\tau^{k}}{k!}\cdot \sum_{n=k}^{\infty }f^{\left(n\right)}\left(t_{n}\right)p_{nk}\right)$$

Rearrangement of the indexes *k* and *n* gives the final form of the U-expansion:

(6) $$f\left(t_{0}+\tau \right)=f\left(t_{0}\right)+\sum_{n=1}^{\infty }\left(f^{\left(n\right)}\left(t_{n}\right)\cdot \sum_{k=1}^{n}\frac{\tau^{k}}{k!}p_{nk}\right)$$

### 2.5. The Coefficients

Comparison of Equation (5) with Equations (2)–(4) highlights that for n=k, all the $p_{nk}$ coefficients ($p_{11},\;p_{22}$, …) are equal to 1:

(7) $$p_{nk}=1\;\;\;\;\;\;\;\;\;\;\;\;\;\;\;\;\;\;\;\;\;\;\;\;\;\;\;\;\;\;\;\;\;\;\;\;\;\;\;\;\;\;\;\;\;\;\;\;\;\;\;\;\;\;\;\;\;\;\;\;\;\;\;\;\;\;\;\;\;\;\left(n=k\right)$$

For n=k+1, the $p_{nk}$ coefficients ($p_{21},\;p_{32}$, …) are equal to $t_{0}-t_{k}$, and can be written as

(8) $$p_{nk}=t_{0}-t_{k}=\frac{{\left(t_{0}-t_{k}\right)}^{n-k}}{\left(n-k\right)!}\;\;\;\;\;\;\;\;\;\;\;\;\;\;\;\;\;\;\;\;\;\;\;\;\;\;\;\;\;\;\left(n=k+1\right)$$

For n=k+2, k+3, and k+4, one has

$$\begin{array}{ccc} p_{nk}=\frac{{\left(t_{0}-t_{k}\right)}^{2}}{2!}+ & \left(t_{0}-t_{k}\right)\left(t_{k}-t_{k+1}\right)=\frac{{\left(t_{0}-t_{k}\right)}^{n-k}}{\left(n-k\right)!}+\sum_{i_{1}=k+1}^{n-1}\frac{{\left(t_{0}-t_{k}\right)}^{i_{1}-k}}{\left(i_{1}-k\right)!}\frac{{\left(t_{k}-t_{i_{1}}\right)}^{n-i_{1}}}{\left(n-i_{1}\right)!} & \left(n=k+2\right)\\ p_{nk}=\frac{{\left(t_{0}-t_{k}\right)}^{3}}{3!}+ & \left(\left(t_{0}-t_{k}\right)\frac{{\left(t_{k}-t_{k+1}\right)}^{2}}{2!}+\frac{{\left(t_{0}-t_{k}\right)}^{2}}{2!}\left(t_{k}-t_{k+2}\right)\right)+\left(t_{0}-t_{k}\right)\left(t_{k}-t_{k+1}\right)\left(t_{k+1}-t_{k+2}\right) & \\ & =\frac{{\left(t_{0}-t_{k}\right)}^{n-k}}{\left(n-k\right)!}+\sum_{i_{1}=k+1}^{n-1}\frac{{\left(t_{0}-t_{k}\right)}^{i_{1}-k}}{\left(i_{1}-k\right)!}\frac{{\left(t_{k}-t_{i_{1}}\right)}^{n-i_{1}}}{\left(n-i_{1}\right)!} & \\ & +\sum_{i_{1}=k+1}^{n-2}\sum_{i_{2}=i_{1}+1}^{n-1}\frac{{\left(t_{0}-t_{k}\right)}^{i_{1}-k}}{\left(i_{1}-k\right)!}\frac{{\left(t_{k}-t_{i_{1}}\right)}^{i_{2}-i_{1}}}{\left(i_{2}-i_{1}\right)!}\frac{{\left(t_{i_{1}}-t_{i_{2}}\right)}^{n-i_{2}}}{\left(n-i_{2}\right)!} & \left(n=k+3\right)\\ p_{nk}=\frac{{\left(t_{0}-t_{k}\right)}^{4}}{4!}+ & \left(\left(t_{0}-t_{k}\right)\frac{{\left(t_{k}-t_{k+1}\right)}^{3}}{3!}+\frac{{\left(t_{0}-t_{k}\right)}^{2}}{2!}\frac{{\left(t_{k}-t_{k+2}\right)}^{2}}{2!}+\frac{{\left(t_{0}-t_{k}\right)}^{3}}{3!}\left(t_{k}-t_{k+3}\right)\right) & \\ & +(\left(t_{0}-t_{k}\right)\left(t_{k}-t_{k+1}\right)\frac{{\left(t_{k+1}-t_{k+2}\right)}^{2}}{2!}+\left(t_{0}-t_{k}\right)\frac{{\left(t_{k}-t_{k+1}\right)}^{2}}{2!}\left(t_{k+1}-t_{k+3}\right)+\frac{{\left(t_{0}-t_{k}\right)}^{2}}{2!}(t_{k} & \\ & -t_{k+2})\left(t_{k+2}-t_{k+3}\right))+\left(t_{0}-t_{k}\right)\left(t_{k}-t_{k+1}\right)\left(t_{k+1}-t_{k+2}\right)\left(t_{k+2}-t_{k+3}\right) & \\ & =\frac{{\left(t_{0}-t_{k}\right)}^{n-k}}{\left(n-k\right)!}+\sum_{i_{1}=k+1}^{n-1}\frac{{\left(t_{0}-t_{k}\right)}^{i_{1}-k}}{\left(i_{1}-k\right)!}\frac{{\left(t_{k}-t_{i_{1}}\right)}^{n-i_{1}}}{\left(n-i_{1}\right)!} & \\ & +\sum_{i_{1}=k+1}^{n-2}\sum_{i_{2}=i_{1}+1}^{n-1}\frac{{\left(t_{0}-t_{k}\right)}^{i_{1}-k}}{\left(i_{1}-k\right)!}\frac{{\left(t_{k}-t_{i_{1}}\right)}^{i_{2}-i_{1}}}{\left(i_{2}-i_{1}\right)!}\frac{{\left(t_{i_{1}}-t_{i_{2}}\right)}^{n-i_{2}}}{\left(n-i_{2}\right)!} & \\ & +\sum_{i_{1}=k+1}^{n-3}\sum_{i_{2}=i_{1}+1}^{n-2}\sum_{i_{3}=i_{2}+1}^{n-1}\frac{{\left(t_{0}-t_{k}\right)}^{i_{1}-k}}{\left(i_{1}-k\right)!}\frac{{\left(t_{k}-t_{i_{1}}\right)}^{i_{2}-i_{1}}}{\left(i_{2}-i_{1}\right)!}\frac{{\left(t_{i_{1}}-t_{i_{2}}\right)}^{i_{3}-i_{2}}}{\left(i_{3}-i_{2}\right)!}\frac{{\left(t_{i_{2}}-t_{i_{3}}\right)}^{n-i_{3}}}{\left(n-i_{3}\right)!} & \left(n=k+4\right) \end{array}$$

The other $p_{nk}$ coefficients (n>k+4) are defined by the following general equation:

$$\begin{array}{c} p_{nk}\\ =\frac{{\left(t_{0}-t_{k}\right)}^{n-k}}{\left(n-k\right)!}+\sum_{i_{1}=k+1}^{n-1}\frac{{\left(t_{0}-t_{k}\right)}^{i_{1}-k}}{\left(i_{1}-k\right)!}\frac{{\left(t_{k}-t_{i_{1}}\right)}^{n-i_{1}}}{\left(n-i_{1}\right)!}+\sum_{i_{1}=k+1}^{n-2}\sum_{i_{2}=i_{1}+1}^{n-1}\frac{{\left(t_{0}-t_{k}\right)}^{i_{1}-k}}{\left(i_{1}-k\right)!}\frac{{\left(t_{k}-t_{i_{1}}\right)}^{i_{2}-i_{1}}}{\left(i_{2}-i_{1}\right)!}\frac{{\left(t_{i_{1}}-t_{i_{2}}\right)}^{n-i_{2}}}{\left(n-i_{2}\right)!}\\ +\sum_{i_{1}=k+1}^{n-3}\sum_{i_{2}=i_{1}+1}^{n-2}\sum_{i_{3}=i_{2}+1}^{n-1}\frac{{\left(t_{0}-t_{k}\right)}^{i_{1}-k}}{\left(i_{1}-k\right)!}\frac{{\left(t_{k}-t_{i_{1}}\right)}^{i_{2}-i_{1}}}{\left(i_{2}-i_{1}\right)!}\frac{{\left(t_{i_{1}}-t_{i_{2}}\right)}^{i_{3}-i_{2}}}{\left(i_{3}-i_{2}\right)!}\frac{{\left(t_{i_{2}}-t_{i_{3}}\right)}^{n-i_{3}}}{\left(n-i_{3}\right)!}+\cdots \\ +\sum_{i_{1}=k+1}^{n-\left(n-k-1\right)}\sum_{i_{2}=i_{1}+1}^{n-\left(n-k-2\right)}\ldots \sum_{i_{n-k-1}=i_{n-k-2}+1}^{n-1}\frac{{\left(t_{0}-t_{k}\right)}^{i_{1}-k}}{\left(i_{1}-k\right)!}\frac{{\left(t_{k}-t_{i_{1}}\right)}^{i_{2}-i_{1}}}{\left(i_{2}-i_{1}\right)!}\frac{{\left(t_{i_{1}}-t_{i_{2}}\right)}^{i_{3}-i_{2}}}{\left(i_{3}-i_{2}\right)!}\cdots \frac{{\left(t_{i_{n-k-2}}-t_{i_{n-k-1}}\right)}^{n-i_{n-k-1}}}{\left(n-i_{n-k-1}\right)!} \end{array}$$

Assuming $t_{i_{-1}}\equiv t_{0}$, $\;i_{0}\equiv k$, $t_{i_{0}}\equiv t_{k}$, $i_{j+1}\equiv n$, and $t_{\;i_{j+1}}\equiv t_{n}$, the coefficients $p_{nk}$ with n≥k+2 can also be reduced to

(9) $$p_{nk}=\frac{{\left(t_{0}-t_{k}\right)}^{n-k}}{\left(n-k\right)!}+\sum_{j=1}^{n-k-1}\left[\sum_{i_{1}=k+1}^{n-j}\;\;\sum_{i_{2}=i_{1}+1}^{n-j+1}\cdots \sum_{i_{j}=i_{j-1}+1}^{n-1}\left(\prod_{m=0}^{j}\frac{{\left(t_{i_{m-1}}-t_{i_{m}}\right)}^{i_{m+1}-i_{m}}}{\left(i_{m+1}-i_{m}\right)!}\right)\right]$$

The Appendix displays the U-expansion with terms up to the 6th order and explicit expression of the $p_{nk}\;$coefficients (Equation (A1)).

### 2.6. A Specific Example of U-Expansion

In this section, we consider a specific type of U-expansion defined by the condition that the derivatives $f^{\left(n\right)}$ of f are detected at progressively earlier times $t_{n}$ as the derivative order *n* increases. Specifically, given an arbitrary time increment t>0, the time instants $t_{n}$ are defined by the following condition:

(10) $$t_{n}=t_{0}-nt\;\;\;\;\;\;\;\;\;\;\;\;\;\;\;\;\left(n=1,\;2,\;\ldots \right)$$

Substitution of Equation (10) into Equations (6)–(9) yields

(11) $$f\left(t_{0}+\tau \right)=f\left(t_{0}\right)+\sum_{n=1}^{\infty }\left(f^{\left(n\right)}\left(t_{0}-nt\right)\sum_{k=1}^{n}\frac{\tau^{k}}{k!}c_{nk}t^{n-k}\right)$$

with the numerical coefficient $c_{nk}$ given by $\left(i_{-1}=0,i_{0}=k,\;i_{j+1}=n\right)$:

(12) $$c_{nk}=1\;\;\;\;\;\;\;\;\;\;\;\;\;\;\;\;\;\;\;\;\;\;\;\;\;\;\;\;\;\;\;\;\;\;\;\;\;\;\;\;\;\;\;\;\;\;\;\;\;\left(k=n\right)$$

(13) $$c_{nk}=k=\frac{k^{\left(n-k\right)}}{\left(n-k\right)!}\;\;\;\;\;\;\;\;\;\;\;\;\;\;\;\;\;\;\;\;\;\;\;\;\;\;\;\;\;\;\left(k=n-1\right)$$

(14) $$c_{nk}=\frac{k^{n-k}}{\left(n-k\right)!}+\sum_{j=1}^{n-k-1}\left(\sum_{i_{1}=k+1}^{n-j}\;\;\sum_{i_{2}=i_{1}+1}^{n-j+1}\cdots \sum_{i_{j}=i_{j-1}+1}^{n-1}\left(\prod_{m=0}^{j}\frac{{\left(i_{m}-i_{m-1}\right)}^{i_{m+1}-i_{m}}}{\left(i_{m+1}-i_{m}\right)!}\right)\right)\;\;\;\;\;\;\;\;\;\;\;\;\;\;\;\;\;\;\;\;\;\left(k\leq n-2\right)$$

The numerical value of the $c_{nk}$ coefficients can be readily calculated from the above equations, yielding the final expression of the expansion reported in the Appendix, where terms up to the 9th-order time derivative have been included (Equation (A2)). 

In the next section, the error related to the truncation of the U-expansions (Equation (A2)) will be calculated for different values of t and τ and for different orders of truncation $n_{t}$, and will also be compared with the truncation error of the corresponding Taylor series (t=0).

## 3. Results

In the following, we assume that time, and consequently the time instant $t_{0}$, and the time increments τ and t are normalized to an arbitrary time constant, so that they can be considered dimensionless quantities. Thus, consistency of notation with Equations (1)–(14) can be maintained in the Taylor series of the exponential and sine functions:

(15) $$e^{t_{0}+\tau }=e^{t_{0}}+e^{t_{0}}\cdot \frac{\tau }{1!}+e^{t_{0}}\cdot \frac{\tau^{2}}{2!}+e^{t_{0}}\cdot \frac{\tau^{3}}{3!}+e^{t_{0}}\cdot \frac{\tau^{4}}{4!}+\ldots$$

(16) $$\sin\left(t_{0}+\tau \right)=\sin\left(t_{0}\right)+\cos\left(t_{0}\right)\cdot \frac{\tau }{1!}-\sin\left(t_{0}\right)\cdot \frac{\tau^{2}}{2!}-\cos\left(t_{0}\right)\cdot \frac{\tau^{3}}{3!}+\sin\left(t_{0}\right)\cdot \frac{\tau^{4}}{4!}+\ldots$$

and in the corresponding U-expansions (11):

(17) $$\begin{array}{c} e^{t_{0}+\tau }=e^{t_{0}}+e^{\left(t_{0}-t\right)}\frac{\tau }{1!}+e^{\left(t_{0}-2t\right)}\left[\frac{\tau^{2}}{2!}+\left(t\right)\tau \right]+e^{\left(t_{0}-3t\right)}\left[\frac{\tau^{3}}{3!}+\left(2t\right)\frac{\tau^{2}}{2!}+\left(\frac{3}{2}t^{2}\right)\tau \right]\\ \;+e^{\left(t_{0}-4t\right)}\left[\frac{\tau^{4}}{4!}+\left(3t\right)\frac{\tau^{3}}{3!}+\left(4t^{2}\right)\frac{\tau^{2}}{2!}+\left(\frac{8}{3}t^{3}\right)\tau \right]+\ldots \end{array}$$

(18) $$\begin{array}{c} \sin\left(t_{0}+\tau \right)=\sin\left(t_{0}\right)+\cos\left(t_{0}-t\right)\cdot \frac{\tau }{1!}-\sin\left(t_{0}-2t\right)\left[\frac{\tau^{2}}{2!}+\left(t\right)\tau \right]-\cos\left(t_{0}-3t\right)\left[\frac{\tau^{3}}{3!}+\left(2t\right)\frac{\tau^{2}}{2!}+\left(\frac{3}{2}t^{2}\right)\tau \right]\\ \;+\sin\left(t_{0}-4t\right)\left[\frac{\tau^{4}}{4!}+\left(3t\right)\frac{\tau^{3}}{3!}+\left(4t^{2}\right)\frac{\tau^{2}}{2!}+\left(\frac{8}{3}t^{3}\right)\tau \right]+\ldots \end{array}$$

**Figure 1:**
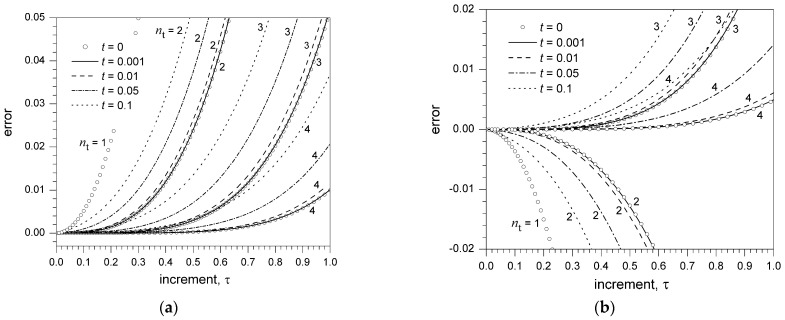
(**a**) Dependence on the increment τ of the error $\varepsilon_{U,n_{t}}$ related to the truncation of the U-expansion of $e^{t_{0}+\tau }$ (Equation (17)) for $t_{0}=0$, different values of t (t=0.001, 0.01, 0.05, 0.1), and different orders of truncation $n_{t}$ $\left(n_{t}=2,\;3,\;4\right)$. The error $\varepsilon_{T,n_{t}}$ related to the truncation $\left(n_{t}=1,\;2,\;3,4\right)$ of the corresponding Taylor series (Equation (15)) is also reported for comparison. (**b**) Dependence on the increment τ of the error $\varepsilon_{U,n_{t}}$ related to the truncation of the U-expansion of $\sin(t_{0}+\tau )$ (Equation (18)) for $t_{0}=\pi /4$, different values of t (t=0.001, 0.01, 0.05, 0.1), and different orders of truncation $n_{t}$ $\left(n_{t}=2,\;3,\;4\right)$. The error $\varepsilon_{T,n_{t}}$ related to the truncation $\left(n_{t}=1,\;2,\;3,4\right)$ of the corresponding Taylor series (Equation (16)) is also reported for comparison. The error is defined as the difference between the exact value of the function and the value of the truncated series expansion.

**Figure 2:**
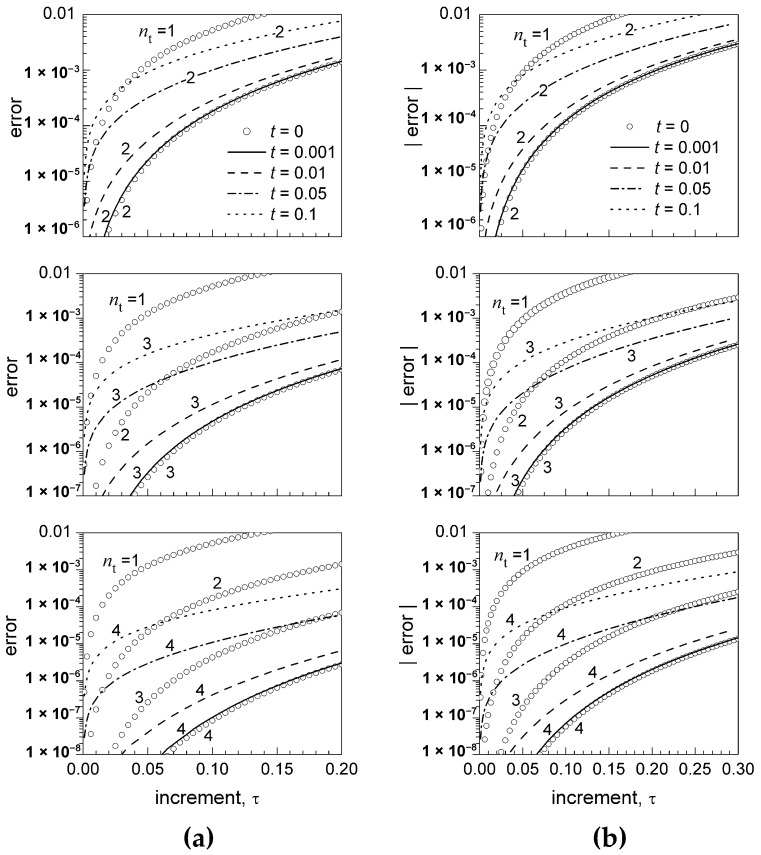
(**a**) Detail of Figure 1a in the range 0<τ≤0.2; (**b**) detail of Figure 1b in the range 0<τ≤0.3.

Figure 1 and Figure 2 display, for different values of t (t=0.001, 0.01, 0.05, 0.1) and different orders of truncation $n_{t}$ $\left(n_{t}=2,\;3,\;4\right)$, the dependence on the increment τ of the error $\varepsilon_{U,n_{t}}$ related to the truncation of the exponential U-expansion (Equation (17)) about $t_{0}=0$ (Figure 1a and Figure 2a) and the sine U-expansion (Equation (18)) about $t_{0}=\pi /4$ (Figure 1b and Figure 2b). For comparison, the figures also display the error $\varepsilon_{T,n_{t}}$ related to the truncation $\left(n_{t}=1,\;2,\;3,4\right)$ of the corresponding Taylor series expansions (15) and (16) about $t_{0}=0$ and $t_{0}=\pi /4$, respectively. 

For τ≫t, the error of the U-expansion is nearly coincident with the error of the corresponding Taylor series with the same order of truncation $\left(\varepsilon_{U,n_{t}}=\varepsilon_{T,n_{t}}\right)$, as long as t≤0.001 (Figure 1). Conversely, with a gradual increase of t from 0.001 to 0.1, the error of the U-expansion becomes progressively higher than the error of the corresponding Taylor series with the same order of truncation $\left(\varepsilon_{U,n_{t}}>\varepsilon_{T,n_{t}}\right)$. However, still with t=0.01 and t=0.05, the error of the U-expansion truncated at order $n_{t}$ remains considerably smaller than the error of the corresponding Taylor series truncated at order $n_{t}-1$ $\left(\varepsilon_{U,n_{t}}<\varepsilon_{T,n_{t}-1}\right)$. 

For τ≈t or τ<t, with a progressive decrease in τ and increase in t, the error $\varepsilon_{U,n_{t}}$ tends to drift towards the error of the Taylor series truncated at progressively lower orders (Figure 2). For example, for the exponential function, $\varepsilon_{U,3}=\varepsilon_{T,2}$ when t=τ=0.05, whereas $\varepsilon_{U,4}=\varepsilon_{T,2}$ when t=0.1 and τ = 0.05 (Figure 2a). Nevertheless, for small values of *t* (t≤0.01) and 0<τ≤t, the error $\varepsilon_{U,n_{t}}\;$becomes negligible, yet at the second-order $n_{t}$ of truncation ($\varepsilon_{U,n_{t}}$ is smaller than about 10^−5^, 10^−7^, and 10^−9^ for $n_{t}=2,\;3,$ and 4, respectively). A nearly identical trend is displayed by the sine function (Figure 2b). 

## 4. Discussion

We have derived a power series expansion (named the U-expansion) to predict the time evolution of a function f given the value $f\left(t_{0}\right)$ of the function at a time instant $t_{0}$ and the values $f^{\left(n\right)}\left(t_{n}\right)$ of the derivatives $f^{\left(n\right)}$ of f at arbitrarily different times $t_{n}$ (*n* = 1, 2, …). The U-expansion can potentially constitute the analytical background for the development of numerical algorithms designed for the fast and accurate feedback control of nonlinear systems. This relies on the possibility that the controlled process variable f and its derivatives can be directly measured. Indeed, when only the controlled process variable f is recorded over time with a given sampling period *T*, all the derivatives $f^{\left(n\right)}$ can be estimated, at the same time instant $t_{0}$ and at any order of approximation, from the recorded values of the variable f, by means of backward finite difference approximations [24]. The standard Taylor series (1) can then be used to estimate the $f\left(t_{0}+\tau \right)$ value. However, the *n*th-order backward finite difference approximation to the *n*th-order derivative $f^{\left(n\right)}\left(t_{0}\right)$ requires preventive recording of 2*n* values of the function f at times $t_{0}\;$, $t_{0}-T$,$\;t_{0}-2T$, …, $t_{0}-\left(2n-1\right)T$. With this degree of approximation, prediction of the time evolution of the variable f can only be attained after a time lag (2n−1)T. Moreover, further processing and delay is needed to avoid noise amplification due to differentiation. 

The sensorimotor system is provided by a variety of different sensory receptors that can directly detect not only the intensity of a stimulus (slow-adapting receptors), but also its rate of change and the higher-order time derivatives (fast-adapting receptors). The slow-adapting receptors also exhibit the adaptation phenomenon: the continuous persistence of a constant stimulus is accompanied by a progressive decrease in the discharge frequency of the afferent sensory neuron. This reflects the fact that in nature, it is more important to feel the change of a condition rather than to be continuously informed that the condition has not changed. Direct measurement of higher-order derivatives, with no delay for their approximate computation, enables the sensorimotor system to make more accurate and fast predictions, and ultimately, optimal efferent responses acting with minimum latency. Nevertheless, the fundamental property of the central nervous system that only relevant sensory information (including higher-order derivatives) is readily transmitted to the integration and processing centers has the drawback that afferent information originating from different sensory receptors is not synchronized in time. 

The U-expansion actually allows the prediction of the time evolution of a function from the values of the function and its time derivatives detected at different times, with no delay for computation. Thus, the U-expansion can be used to optimize the feedback control of a physiological parameter *f*, enabling, at the same time, a high degree of accuracy and the minimization of the latency of the control response. Ultimately, the U-expansion constitutes an unanalytical framework to simulate the fast and accurate prediction strategy of the sensorimotor system. 

The U-expansion has been numerically applied to the ideal problem of the calculation of $f\left(t_{0}+\tau \right)$ from the value $f\left(t_{0}\right)$ and the values of the derivatives $f^{\left(n\right)}$ detected at progressively earlier times $t_{n}=t_{0}-nt\;(t>0)$ as the derivative order *n* increases (n = 1, 2, …). This particular case is of relevance when accurate values of lower-order derivatives may be retrieved earlier, compared to higher-order derivatives, which typically require a more demanding detection process. For sufficiently small values of t and increments of τ significantly greater than t, the error $\varepsilon_{U,n_{t}}$ associated with the truncation of the U-expansion at a given order $n_{t}$ closely equalizes the error $\varepsilon_{T,n_{t}}$ of the corresponding Taylor series (t=0) truncated at the same order (Figure 1). Small values of the t increment and higher values of the τ increment actually correspond to the high-frequency discharge of sensory neurons and the need for longer-term prediction, respectively. With a progressive increase in t, the error $\varepsilon_{U,n_{t}}$ associated with the truncation of the U-expansion at order $n_{t}$ drifts towards the error of the corresponding Taylor series truncated at progressively lower orders (Figure 1 and Figure 2). Nevertheless, $n_{t}$ can be progressively increased to render $\varepsilon_{U,n_{t}}$ equal to the error of the Taylor series truncated at a specific lower order, with no increase in the delay for the calculation of $f\left(t_{0}+\tau \right)$.

Although the previous findings have been derived for a particular regular set of detection times $t_{n}$ ($t_{n}=t_{0}-nt$), they nevertheless have a broad validity. Indeed, the same findings also result from any arbitrary set of detection times $t_{n}$, provided t represents the maximum value among the differences $\left|t_{i}-t_{i-1}\right|$ (i = 1, 2, …) relative to the detection times of two successive derivatives. Among these infinite sets of detection times, that defined by the selected condition $(t_{n}=t_{0}-nt$) clearly corresponds to the most unfavorable condition for making predictions.

The present study provides a general analytical algorithm for the simulation of the fast prediction strategy of the sensorimotor system. Detailed neurophysiological models are needed when the specific organizational principles and coding mechanisms of the sensorimotor system are considered. Indeed, the incoming stream of sensory signals in the CNS is encoded as trains of action potentials; processed in stages in the sequential relay nuclei of the spinal cord, brain stem, thalamus, and cerebral cortex; and modulated by superspinal centers through descending pathways, according to its current (context-dependent) functional relevance [4,18,25,26]. The rigorous experimental investigation of the neuronal mechanisms underlying the fast prediction strategy of the sensorimotor system was beyond the scope of this study. Nonetheless, this study unveils, for the first time, a seminal plausible algorithm (U-expansion) that enables fast and accurate sensorimotor predictions and accounts for the experimental evidence that the sensory receptors can collectively sense the unsynchronized values of a physiological parameter and its time derivatives.

## 5. Conclusions

The U-expansion derived in this paper determines the time evolution of a physiological parameter from the value of the parameter and the values of its successive time derivatives detected at different times by different sensory receptors. This new series expansion constitutes a generalization of the Taylor series that potentially provides an analytical background for the study of the fast prediction strategies of the sensorimotor system, as well as for the development of numerical algorithms designed for the feedback control of nonlinear systems. 

## Data Availability

Not applicable.

## Appendix A

The following equation constitutes the explicit expression of the U-expansion, including terms up to the 6th order:

(A1) $$\begin{array}{cc} f\left(t_{0}+\tau \right)=f\left(t_{0}\right) & +f^{\left(1\right)}\left(t_{1}\right)\tau +f^{\left(2\right)}\left(t_{2}\right)\left\{\frac{\tau^{2}}{2!}+\tau \left(t_{0}-t_{1}\right)\right\}\\ & +f^{\left(3\right)}\left(t_{3}\right)\left\{\frac{\tau^{3}}{3!}+\frac{\tau^{2}}{2!}\left(t_{0}-t_{2}\right)+\tau \left[\frac{{\left(t_{0}-t_{1}\right)}^{2}}{2!}+\left(t_{0}-t_{1}\right)\left(t_{1}-t_{2}\right)\right]\right\}\\ & +f^{\left(4\right)}\left(t_{4}\right)\{\frac{\tau^{4}}{4!}+\frac{\tau^{3}}{3!}\left(t_{0}-t_{3}\right)+\frac{\tau^{2}}{2!}\left[\frac{{\left(t_{0}-t_{2}\right)}^{2}}{2!}+\left(t_{0}-t_{2}\right)\left(t_{2}-t_{3}\right)\right]\\ & +\tau \left[\frac{{\left(t_{0}-t_{1}\right)}^{3}}{3!}+\left(\left(t_{0}-t_{1}\right)\frac{{\left(t_{1}-t_{2}\right)}^{2}}{2!}+\frac{{\left(t_{0}-t_{1}\right)}^{2}}{2!}\left(t_{1}-t_{3}\right)\right)+\left(t_{0}-t_{1}\right)\left(t_{1}-t_{2}\right)\left(t_{2}-t_{3}\right)\right]\}\\ & +f^{\left(5\right)}\left(t_{5}\right)\{\frac{\tau^{5}}{5!}+\frac{\tau^{4}}{4!}\left(t_{0}-t_{4}\right)+\frac{\tau^{3}}{3!}\left[\frac{{\left(t_{0}-t_{3}\right)}^{2}}{2!}+\left(t_{0}-t_{3}\right)\left(t_{3}-t_{4}\right)\right]\\ & +\frac{\tau^{2}}{2!}\left[\frac{{\left(t_{0}-t_{2}\right)}^{3}}{3!}+\left(\left(t_{0}-t_{2}\right)\frac{{\left(t_{2}-t_{3}\right)}^{2}}{2!}+\frac{{\left(t_{0}-t_{2}\right)}^{2}}{2!}\left(t_{2}-t_{4}\right)\right)+\left(t_{0}-t_{2}\right)\left(t_{2}-t_{3}\right)\left(t_{3}-t_{4}\right)\right]\\ & +\tau [\frac{{\left(t_{0}-t_{1}\right)}^{4}}{4!}+\left(\left(t_{0}-t_{1}\right)\frac{{\left(t_{1}-t_{2}\right)}^{3}}{3!}+\frac{{\left(t_{0}-t_{1}\right)}^{2}}{2!}\frac{{\left(t_{1}-t_{3}\right)}^{2}}{2!}+\frac{{\left(t_{0}-t_{1}\right)}^{3}}{3!}\left(t_{1}-t_{4}\right)\right)\\ & +\left(\left(t_{0}-t_{1}\right)\left(t_{1}-t_{2}\right)\frac{{\left(t_{2}-t_{3}\right)}^{2}}{2!}+\left(t_{0}-t_{1}\right)\frac{{\left(t_{1}-t_{2}\right)}^{2}}{2!}\left(t_{2}-t_{4}\right)+\frac{{\left(t_{0}-t_{1}\right)}^{2}}{2!}\left(t_{1}-t_{3}\right)\left(t_{3}-t_{4}\right)\right)\\ & +\left(t_{0}-t_{1}\right)\left(t_{1}-t_{2}\right)\left(t_{2}-t_{3}\right)\left(t_{3}-t_{4}\right)]\}\\ & +f^{\left(6\right)}\left(t_{6}\right)\{\frac{\tau^{6}}{6!}+\frac{\tau^{5}}{5!}\left(t_{0}-t_{5}\right)+\frac{\tau^{4}}{4!}\left[\frac{{\left(t_{0}-t_{4}\right)}^{2}}{2!}+\left(t_{0}-t_{4}\right)\left(t_{4}-t_{5}\right)\right]\\ & +\frac{\tau^{3}}{3!}\left[\frac{{\left(t_{0}-t_{3}\right)}^{3}}{3!}+\left(\left(t_{0}-t_{3}\right)\frac{{\left(t_{3}-t_{4}\right)}^{2}}{2}+\frac{{\left(t_{0}-t_{3}\right)}^{2}}{2!}\left(t_{3}-t_{5}\right)\right)+\left(t_{0}-t_{3}\right)\left(t_{3}-t_{4}\right)\left(t_{4}-t_{5}\right)\right]\\ & +\frac{\tau^{2}}{2!}[\frac{{\left(t_{0}-t_{2}\right)}^{4}}{4!}+\left(\left(t_{0}-t_{2}\right)\frac{{\left(t_{2}-t_{3}\right)}^{3}}{3!}+\frac{{\left(t_{0}-t_{2}\right)}^{2}}{2!}\frac{{\left(t_{2}-t_{4}\right)}^{2}}{2!}+\frac{{\left(t_{0}-t_{2}\right)}^{3}}{3!}\left(t_{2}-t_{5}\right)\right)\\ & +\left(\left(t_{0}-t_{2}\right)\left(t_{2}-t_{3}\right)\frac{{\left(t_{3}-t_{4}\right)}^{2}}{2!}+\left(t_{0}-t_{2}\right)\frac{{\left(t_{2}-t_{3}\right)}^{2}}{2!}\left(t_{3}-t_{5}\right)+\frac{{\left(t_{0}-t_{2}\right)}^{2}}{2!}\left(t_{2}-t_{4}\right)\left(t_{4}-t_{5}\right)\right)\\ & +\left(t_{0}-t_{2}\right)\left(t_{2}-t_{3}\right)\left(t_{3}-t_{4}\right)\left(t_{4}-t_{5}\right)]\\ & +\tau [\frac{{\left(t_{0}-t_{1}\right)}^{5}}{5!}\\ & +\left(\left(t_{0}-t_{1}\right)\frac{{\left(t_{1}-t_{2}\right)}^{4}}{4!}+\frac{{\left(t_{0}-t_{1}\right)}^{2}}{2!}\frac{{\left(t_{1}-t_{3}\right)}^{3}}{3!}+\frac{{\left(t_{0}-t_{1}\right)}^{3}}{3!}\frac{{\left(t_{1}-t_{4}\right)}^{2}}{2!}+\frac{{\left(t_{0}-t_{1}\right)}^{4}}{4!}\left(t_{1}-t_{5}\right)\right)\\ & +(\left(t_{0}-t_{1}\right)\left(t_{1}-t_{2}\right)\frac{{\left(t_{2}-t_{3}\right)}^{3}}{3!}+\left(t_{0}-t_{1}\right)\frac{{\left(t_{1}-t_{2}\right)}^{2}}{2!}\frac{{\left(t_{2}-t_{4}\right)}^{2}}{2!}+\left(t_{0}-t_{1}\right)\frac{{\left(t_{1}-t_{2}\right)}^{3}}{3!}\left(t_{3}-t_{5}\right)\\ & +\frac{{\left(t_{0}-t_{1}\right)}^{2}}{2!}\left(t_{1}-t_{3}\right)\frac{{\left(t_{3}-t_{4}\right)}^{2}}{2!}+\frac{{\left(t_{0}-t_{1}\right)}^{2}}{2!}\frac{{\left(t_{1}-t_{3}\right)}^{2}}{2!}\left(t_{3}-t_{5}\right)+\frac{{\left(t_{0}-t_{1}\right)}^{3}}{3!}\left(t_{1}-t_{4}\right)\left(t_{4}-t_{5}\right))\\ & +(\left(t_{0}-t_{1}\right)\left(t_{1}-t_{2}\right)\left(t_{2}-t_{3}\right)\frac{{\left(t_{3}-t_{4}\right)}^{2}}{2!}+\left(t_{0}-t_{1}\right)\left(t_{1}-t_{2}\right)\frac{{\left(t_{2}-t_{3}\right)}^{2}}{2!}\left(t_{3}-t_{5}\right)\\ & +\left(t_{0}-t_{1}\right)\frac{{\left(t_{1}-t_{2}\right)}^{2}}{2!}\left(t_{2}-t_{4}\right)\left(t_{4}-t_{5}\right)+\frac{{\left(t_{0}-t_{1}\right)}^{2}}{2!}\left(t_{1}-t_{3}\right)\left(t_{3}-t_{4}\right)\left(t_{4}-t_{5}\right))\\ & +\left(t_{0}-t_{1}\right)\left(t_{1}-t_{2}\right)\left(t_{2}-t_{3}\right)\left(t_{3}-t_{4}\right)\left(t_{4}-t_{5}\right)]\}+\cdots \end{array}$$

Under the ideal condition that the derivatives $f^{\left(n\right)}$ are detected at regularly earlier times $t_{n}=t_{0}-nt$ as the derivative order *n* increases (t>0), Equation (A1) becomes

(A2) $$\begin{array}{cc} f\left(t_{0}+\tau \right)=f\left(t_{0}\right) & +f^{\left(1\right)}\left(t_{0}-t\right)\tau +f^{\left(2\right)}\left(t_{0}-2t\right)\left[\frac{\tau^{2}}{2!}+\left(t\right)\tau \right]+f^{\left(3\right)}\left(t_{0}-3t\right)\left[\frac{\tau^{3}}{3!}+\left(2t\right)\frac{\tau^{2}}{2!}+\left(\frac{3}{2}t^{2}\right)\tau \right]\\ & +f^{\left(4\right)}\left(t_{0}-4t\right)\left[\frac{\tau^{4}}{4!}+\left(3t\right)\frac{\tau^{3}}{3!}+\left(4t^{2}\right)\frac{\tau^{2}}{2!}+\left(\frac{8}{3}t^{3}\right)\tau \right]\\ & +f^{\left(5\right)}\left(t_{0}-5t\right)\left[\frac{\tau^{5}}{5!}+\left(4t\right)\frac{\tau^{4}}{4!}+\left(\frac{15}{2}t^{2}\right)\frac{\tau^{3}}{3!}+\left(\frac{25}{3}t^{3}\right)\frac{\tau^{2}}{2!}+\left(\frac{125}{24}t^{4}\right)\tau \right]\\ & +f^{\left(6\right)}\left(t_{0}-6t\right)\left[\frac{\tau^{6}}{6!}+\left(5t\right)\frac{\tau^{5}}{5!}+\left(12t^{2}\right)\frac{\tau^{4}}{4!}+\left(18t^{3}\right)\frac{\tau^{3}}{3!}+\left(18t^{4}\right)\frac{\tau^{2}}{2!}+\left(\frac{54}{4}t^{5}\right)\tau \right]\\ & +f^{\left(7\right)}\left(t_{0}-7t\right)\left[\frac{\tau^{7}}{7!}+\left(6t\right)\frac{\tau^{6}}{6!}+\left(\frac{35}{2}t^{2}\right)\frac{\tau^{5}}{5!}+\left(\frac{98}{3}t^{3}\right)\frac{\tau^{4}}{4!}+\left(\frac{343}{8}t^{4}\right)\frac{\tau^{3}}{3!}+\left(\frac{2401}{60}t^{5}\right)\frac{\tau^{2}}{2!}+\left(\frac{16807}{720}t^{6}\right)\tau \right]\\ & +f^{\left(8\right)}\left(t_{0}-8t\right)[\frac{\tau^{8}}{8!}+\left(7t\right)\frac{\tau^{7}}{7!}+\left(24t^{2}\right)\frac{\tau^{6}}{6!}+\left(\frac{160}{3}t^{3}\right)\frac{\tau^{5}}{5!}+\left(\frac{256}{3}t^{4}\right)\frac{\tau^{4}}{4!}+\left(\frac{512}{5}t^{5}\right)\frac{\tau^{3}}{3!}+\left(\frac{4096}{45}t^{6}\right)\frac{\tau^{2}}{2!}\\ & +\left(\frac{16384}{315}t^{7}\right)\tau ]\\ & +f^{\left(9\right)}\left(t_{0}-9t\right)[\frac{\tau^{9}}{9!}+\left(8t\right)\frac{\tau^{8}}{8!}+\left(\frac{63}{2}t^{2}\right)\frac{\tau^{7}}{7!}+\left(81t^{3}\right)\frac{\tau^{6}}{6!}+\left(\frac{1215}{8}t^{4}\right)\frac{\tau^{5}}{5!}+\left(\frac{2187}{10}t^{5}\right)\frac{\tau^{4}}{4!}+\left(\frac{19683}{80}t^{6}\right)\frac{\tau^{3}}{3!}\\ & +\left(\frac{59049}{280}t^{7}\right)\frac{\tau^{2}}{2!}+\left(\frac{531441}{4480}t^{8}\right)\tau ]+\cdots \end{array}$$
